# Underweight was associated with increased mortality in adults with latent tuberculosis infection

**DOI:** 10.3389/fnut.2025.1607507

**Published:** 2025-06-24

**Authors:** Jun-Zhe Liao, Xiao Liu, Min Qi

**Affiliations:** ^1^Department of Respiratory and Critical Care Medicine, Chengdu Fifth People’s Hospital, Chengdu, China; ^2^Department of Geriatric Intensive Care Unit, Sichuan Provincial People’s Hospital, University of Electronic Science and Technology of China, Chengdu, China

**Keywords:** BMI, underweight, latent tuberculosis infection, mortality, RCS

## Abstract

**Objective:**

Latent tuberculosis (TB) infection (LTBI) is a reservoir for active TB. Although body mass index (BMI) predicts LTBI progression and influences active TB outcomes, its association with mortality in LTBI patients remains unclear. We therefore investigated this relationship in a US cohort.

**Research methods & procedures:**

Data from the National Health and Nutrition Examination Survey 2011–2012 was utilized. Survival differences across BMI categories were assessed with Kaplan–Meier curves and multivariable Cox regression. The Restricted Cubic Spline (RCS) analysis modeled the nonlinear relationship between BMI and mortality risk.

**Results:**

Among 700 LTBI participants analyzed, multivariable Cox regression identified underweight individuals as having higher mortality risk than normalweight counterparts (adjusted HR = 2.77, 95% CI 1.06–7.22, *p* = 0.04). No significant mortality associations were observed for obese or overweight participants across both crude and adjusted models (all *p* > 0.05). RCS analysis demonstrated a U-shaped pattern between BMI and mortality, with minimum mortality risk at BMI 27.3 kg/m^2^ (*p* for nonlinearity = 0.0012).

**Conclusion:**

In LTBI adults, underweight status independently predicted increased mortality risk, while overweight or obesity showed no association. RCS analysis confirmed a U-shaped BMI-mortality relationship with optimal survival at 27.3 kg/m^2^.

## Introduction

The global burden of tuberculosis (TB) remains a significant public health challenge, with mortality rates varying across populations and regions (1). Latent TB infection (LTBI), defined by an enduring immune response to *Mycobacterium tuberculosis* antigens without clinical disease, affects approximately 25% of the global population, with an estimated 5–10% progressing to active TB disease (2). This immense scale of LTBI not only fuels ongoing TB transmission but also poses a fundamental challenge to achieving global TB elimination goals.

While the risk factors influencing on TB outcomes have been extensively studied (3–5), the relationship between body mass index (BMI) and mortality in LTBI individuals warrants focused attention. As an established metric of adiposity and nutritional status, BMI demonstrates prognostic value for clinical outcomes across diverse diseases (6–8), indicating its potential utility in assessing systemic resilience during TB infection. Substantial evidence confirms that reduced BMI independently predicts elevated mortality risk in active TB. Retrospective and prospective cohort studies encompassing both drug-sensitive and drug-resistant TB revealed that lower BMI associated with increased mortality and adverse treatment outcomes (4, 9, 10), potentially mediated through nutritional compromise and immune dysfunction (10). Conversely, BMI demonstrated an inverse epidemiological relationship with LTBI prevalence (11), while underweight status accelerates LTBI progression to active disease-though mortality implications specific to LTBI remain inadequately defined.

Elucidating this relationship is paramount. Given that LTBI represents a prolonged state affecting billions, understanding the potential impact of BMI on mortality among LTBI individuals could revolutionize risk stratification. It holds significant potential to inform targeted public health interventions (e.g., nutritional support programs) and refine clinical monitoring strategies for high-risk LTBI individuals, ultimately contributing to reduced TB-related mortality and the success of the WHO’s End TB Strategy.

Therefore, to address this pivotal knowledge gap, this study specifically investigates the association between BMI and all-cause mortality within a well-defined cohort of individuals diagnosed with LTBI.

## Materials and methods

### Study population

The National Health and Nutrition Examination Survey (NHANES) is a comprehensive nationwide cross-sectional study conducted in the United States. Its primary objective is to evaluate the health and nutritional well-being of the US population. The survey involves standardized household interviews and health assessments conducted at mobile examination centers. These assessments encompass physical evaluations and laboratory tests for the collection of pertinent data. NHANES employs a sophisticated stratified, multilevel probability cluster sampling approach, ensuring the inclusion of a highly representative cross-section of the US population. Ethical approval for the NHANES study protocol was granted by the Research Ethics Review Board of the National Center for Health Statistics (NCHS), and all participants provided written informed consent. Detailed information about the NHANES study design and its data is available to the public.^1^

Survival data for individuals with TB were acquired from the National Death Index (NDI)^2^, which is linked to the NHANES surveys and encompasses a repository of more than 100 million death records. The data collection protocol for NHANES surveys has received approval from the NCHS institutional review board. The Ethics Committee has affirmed that the data is analyzable and publishable under a waiver of consent from the individuals involved. Prior to utilization, the data employed in this study underwent anonymization procedures.

We utilized the 2011–2012 NHANES survey cycle for evaluating the correlation between BMI and all-cause mortality among individuals with LTBI. This specific cycle was chosen due to its comprehensive coverage of recent TB testing data for all participants aged ≥ 6 years. To ensure data quality, we excluded participants below the age of 18 and samples without positive QuantiFERON-TB Gold-In-Tube (QFT) or Tuberculin skin test (TST) results.

### Assessment of BMI

Body Mass Index (BMI) was computed by dividing weight in kilograms by the square of height in meters, with rounding to a single decimal place. Adhering to the guidelines set forth by the World Health Organization, the BMI classifications were defined as follows (12): underweight <18.5 kg/m^2^, normalweight 18.5–24.9 kg/m^2^, overweight 25–29.9 kg/m^2^, and obesity ≥30 kg/m^2^.

### The definition of LTBI

To determine the TB infection, NHANES participants underwent a skin test using a commercially available antigen, tuberculin-purified protein derivative (PPD) known as tubersol. A controlled amount of 0.1 mL (5 international units) of the designated PPD was administered. Trained NHANES technicians, who were blinded to the participant’s medical history and any potential TB contact, conducted measurements of reactions between 46 and 76 h later.

Furthermore, NHANES participants underwent a secondary screening through the use of an FDA-approved IGRA blood test, specifically the QFT-GIT, to detect TB infection. The QFT-GIT system employs specialized blood collection tubes, including a Nil control tube, a TB Antigen tube, and a Mitogen tube (positive control), for the collection of whole blood via venipuncture. Following collection, the tubes are agitated to mix the antigen with whole blood and then incubated at 37°C ± 1°C for 16 to 24 h. After the incubation period, plasma is harvested and the ELISA method is used to quantify the amount of IFN-*γ* produced in response to the peptide antigens.

In our study, a positive TST was defined as an induration size of ≥ 10 mm, a criterion commonly used for adults in the US, except for individuals with special risk factors. For the interpretation of a positive QFT-GIT, the following criteria were applied in accordance with NHANES guidelines: (1) The Nil value was required to be ≤ 8.0 IU gamma interferon (IF)/ml, (2) The TB antigen value minus the Nil value had to be ≥ 0.35 IU IF/ml, and (3) The TB antigen value minus the Nil value had to be ≥ 25% of the Nil value. Furthermore, a low response to mitogen (< 0.5 IU/mL) was considered indicative of an indeterminate result when a blood sample also exhibited a negative response to the TB antigens.

Individuals who had a positive result from either the TST or QFT-GIT, and subsequently was ruled out for active TB disease, and possessed complete mortality outcome documentation throughout the study period, were definitively classified as LTBI cases and included in the final analytical cohort.

### Variables

Our analysis took into account various factors, such as age, gender, race, education level, marital status, the presence of chronic kidney diseases (CKD), history of asthma, chronic obstructive pulmonary diseases (COPD), diabetes mellitus (DM), alcohol consumption, smoking status. Moreover, we integrated laboratory measurements into our study, including parameters like white blood cell (WBC) count, lymphocyte count, monocyte count, neutrophil count, hemoglobin level, albumin level, sodium level, and glucose level. DM was defined using multiple criteria, including self-reported DM diagnosis, HbA1c levels ≥6.5%, fasting glucose levels ≥7.0 mmol/L, random blood glucose levels ≥11.1 mmol/L, two-hour plasma glucose levels ≥11.1 mmol/L in an oral glucose tolerance test, or the use of oral hypoglycemic agents or insulin. Comprehensive information concerning these variables can be accessed on the website: https://www.cdc.gov/nchs/nhanes/.

### Statistical analysis

The statistical analysis was conducted adhering to NHANES analysis guidelines, incorporating intricate sampling weights to account for complex multi-stage clustering surveys. Missing data was imputed with Multiple imputation with chained equations. Continuous variables were represented by their mean values along with their corresponding standard deviations, while categorical variables were expressed in terms of counts and proportions. To evaluate differences across BMI-stratified groups, weighted one-way ANOVA with Bonferroni post-hoc testing was employed for continuous variables, while for categorical variables, weighted chi-square test was utilized, supplemented by Fisher’s exact test when expected frequencies were <5.

For the purpose of visualizing the survival differences among distinct BMI groups, a Kaplan–Meier curve was generated. The correlation between divergent BMI groups and the probability of mortality was examined using a multivariable Cox regression model, with meticulous adjustment for potential confounding variables. No covariates were adjusted for in Crude Model. Model 1 adjusted for age, gender, race, and educational level. Model 2 expanded adjustment to include DM, CKD, COPD, and asthma. Finally, Model 3 incorporated all previous covariates plus lymphocyte count.

To explore potential nonlinear relationships within the regression models comprehensively, the Restricted Cubic Spline (RCS) analysis, incorporating four distinct piecewise points, was employed. This method, widely embraced in the realms of epidemiology and clinical trials, facilitated an in-depth assessment of the intricate associations between BMI and the risk of mortality.

All analyses were executed utilizing R version 4.2.2 (https://www.r-project.org/, The R Foundation). A significance threshold of *p* < 0.05 was employed to determine statistical significance.

## Results

### Baseline characteristics of LTBI participants stratified by BMI category

**Figure 1:**
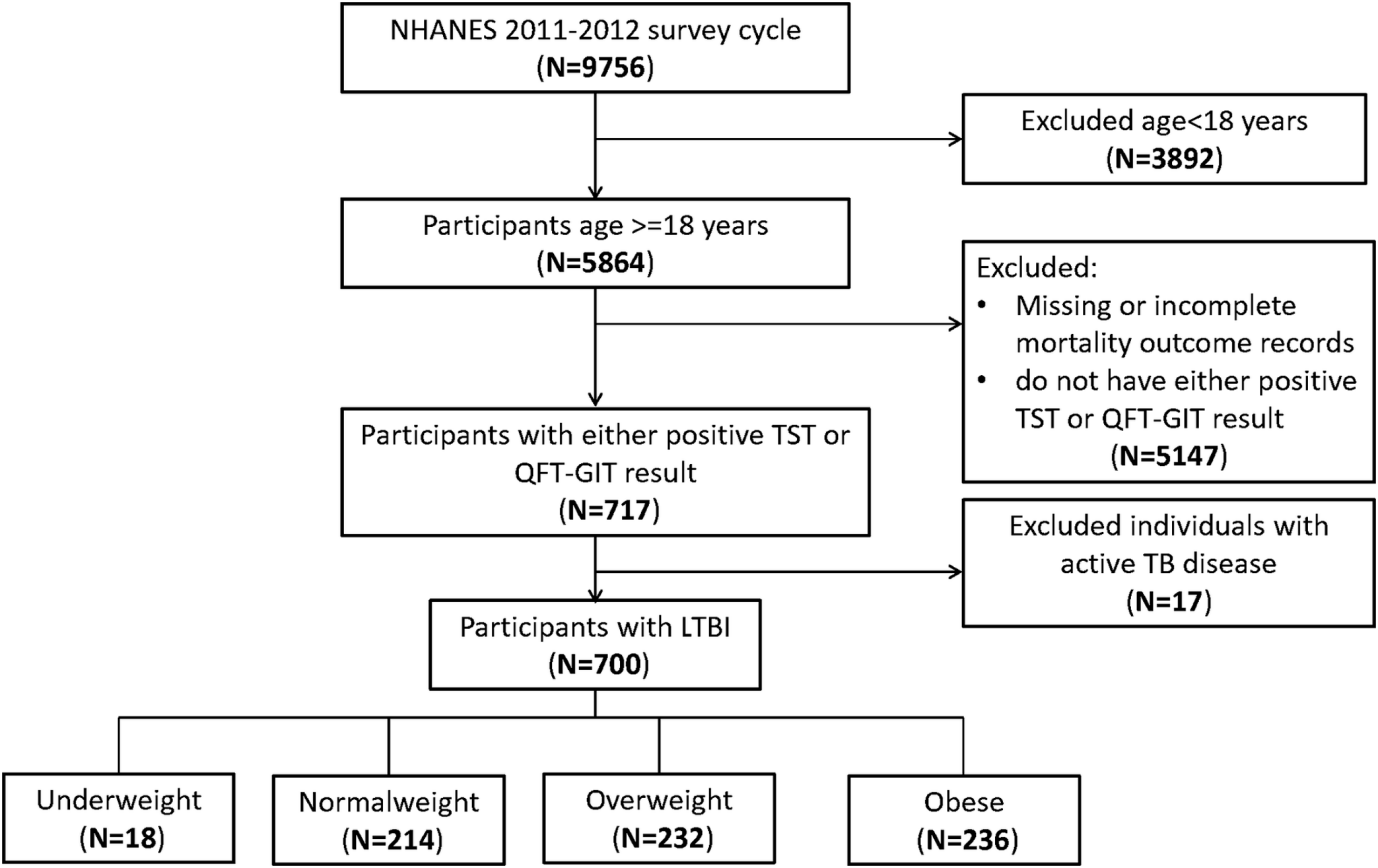
Flowchart of the sample selection from National Health and Nutrition Examination Survey (NHANES) 2011–2012.

At the outset, our study included 9,756 participants. After excluding those aged under 18 (*n* = 3,892), those lacking either a positive TST or QFT-GIT result (*n* = 5,147), and those with active TB disease (*n* = 17), our final analysis comprised 700 participants (Figure 1).

The weighted baseline characteristics of the included individuals were presented in Table 1. Our study involved a total of 700 participants with LTBI, with a mean age of 50.58 ± 0.96 years, among whom 44.80% were female and 55.20% were male. The mean BMI was28.74 ± 0.44 kg/m^2^. TST and QFT-GIT positivity rates were 28.00 and 71.56%, respectively. Analysis across BMI categories revealed statistically significant differences (all *p* < 0.05) in race, educational levels, presence of DM or CKD, WBC count, lymphocyte count, neutrophil count, platelet count, albumin levels, glucose levels. Notably, compared to the normalweight group, underweight and obese participants exhibited increased mortality rates, while overweight participants demonstrated a decreased mortality rate.

**Table 1:** Baseline characteristics of LTBI participants stratified by BMI category.

Variables	All participants (*N* = 700)	Normalweight (*N* = 214)	Obese (*N* = 236)	Overweight (*N* = 232)	Underweight (*N* = 18)	*p* value
Age (years)	50.58 (0.96)	47.20 (2.01)	51.87 (1.24)	51.95 (1.72)	54.86 (6.54)	0.34
BMI (kg/m^2^)	28.74 (0.44)	22.68 (0.13)	35.80 (0.48)	27.32 (0.14)	17.52 (0.16)	**<0.0001**
Gender						0.06
Female	304 (44.80)	89 (41.13)	122 (52.65)	85 (38.49)	8 (59.99)	
Male	396 (55.20)	125 (58.87)	114 (47.35)	147 (61.51)	10 (40.01)	
Race						**0.01**
Mexican American	106 (18.37)	21 (11.66)	55 (27.81)	29 (15.18)	1 (3.52)	
Non-Hispanic Black	165 (14.56)	43 (13.29)	73 (17.89)	44 (11.79)	5 (18.79)	
Non-Hispanic White	88 (31.80)	23 (34.05)	35 (30.73)	26 (30.06)	4 (44.19)	
Others	341 (35.27)	127 (41.00)	73 (23.57)	133 (42.97)	8 (33.50)	
Marital status						0.16
Divorced	69 (9.86)	15 (7.00)	28 (11.86)	25 (10.76)	1 (2.21)	
Living with partner	45 (6.43)	8 (3.74)	21 (8.90)	15 (6.47)	1 (3.94)	
Married	392 (56.00)	130 (60.75)	120 (50.85)	132 (56.90)	10 (59.11)	
Never married	79 (11.29)	31 (14.49)	18 (7.63)	28 (12.07)	2 (13.67)	
Separated	41 (5.86)	10 (1.87)	19 (8.05)	12 (5.17)	0 (0.00)	
Widowed	63 (9.00)	14 (6.54)	28 (11.86)	17 (7.33)	4 (21.06)	
NA	11 (15.71)	6 (5.14)	2 (0.85)	3 (1.29)	0 (0.00)	
Education levels						**0.02**
College graduate or above	153 (24.44)	67 (35.39)	33 (14.71)	48 (24.25)	5 (36.52)	
High school graduate or less	406 (55.98)	99 (43.89)	155 (64.42)	143 (58.63)	9 (44.10)	
Some college or AA degree	141 (19.57)	48 (20.72)	48 (20.87)	41 (17.12)	4 (19.38)	
Alcohol use						0.94
No	119 (17.00)	42 (19.63)	35 (14.83)	40 (17.24)	2 (11.11)	
Yes	491 (70.14)	136 (63.55)	179 (75.85)	165 (71.12)	11 (61.11)	
NA	90 (12.86)	36 (16.82)	22 (9.32)	27 (11.64)	5 (27.78)	
Smoke status						0.88
Former	180 (25.71)	48 (22.43)	70 (29.66)	56 (24.14)	6 (34.53)	
Never	374 (53.43)	120 (56.07)	122 (51.69)	124 (53.45)	8 (46.75)	
Now	135 (19.29)	40 (18.69)	42 (17.80)	49 (21.12)	4 (18.72)	
NA	11 (1.57)	6 (2.80)	2 (0.85)	3 (1.29)	0 (0.00)	
DM						**<0.001**
DM	185 (26.43)	37 (17.29)	95 (40.25)	51 (15.72)	2 (16.54)	
IFG	23 (3.29)	5 (2.34)	7 (2.97)	11 (3.38)	0 (0.00)	
IGT	32 (4.57)	9 (4.21)	16 (6.78)	7 (2.21)	0 (0.00)	
No	459 (65.57)	162 (75.70)	118 (50.00)	163 (78.68)	16 (83.46)	
NA	1 (0.14)	1 (0.47)	0 (0.00)	0 (0.00)	0 (0.00)	
CKD						**0.05**
No	548 (78.29)	173 (80.84)	175 (74.15)	188 (81.03)	12 (66.32)	
Yes	131 (18.71)	31 (14.49)	57 (24.15)	37 (15.95)	6 (33.68)	
NA	21 (3.00)	10 (4.67)	4 (1.70)	7 (3.02)	0 (0.00)	
Asthma						0.22
No	616 (88.45)	197 (93.47)	196 (85.25)	206 (87.65)	17 (84.20)	
Yes	84 (11.55)	17 (6.53)	40 (14.75)	26 (12.35)	1 (15.80)	
COPD						0.76
No	658 (94.00)	199 (92.99)	222 (94.07)	219 (94.40)	18 (100.00)	
Yes	30 (4.29)	9 (4.21)	11 (4.66)	10 (4.31)	0 (0.00)	
NA	12 (1.71)	6 (2.80)	3 (1.27)	3 (1.29)	0 (0.00)	
TST						0.9
Positive	196 (28.00)	60 (28.04)	62 (26.27)	69 (29.74)	5 (27.78)	
Negative	403 (57.57)	123 (57.48)	139 (58.90)	130 (56.03)	11 (61.11)	
NA	101 (14.43)	31 (14.49)	35 (14.84)	33 (14.22)	2 (11.11)	
QFT-GIT						0.93
Indeterminate	3 (0.17)	0 (0.00)	1 (0.15)	2 (0.36)	0 (0.00)	
Negative	197 (28.27)	62 (26.94)	66 (30.01)	63 (27.70)	6 (26.41)	
Positive	500 (71.56)	152 (73.06)	169 (69.84)	167 (71.94)	12 (73.59)	
WBC ^#^	6.92 (0.13)	6.50 (0.28)	6.77 (0.14)	7.44 (0.18)	6.41 (0.38)	**0.005**
Lymphocyte ^#^	2.15 (0.04)	2.05 (0.09)	2.09 (0.06)	2.31 (0.06)	1.75 (0.10)	**<0.001**
Monocyte ^#^	0.51 (0.01)	0.50 (0.02)	0.50 (0.02)	0.54 (0.02)	0.50 (0.04)	0.46
Neutrophils ^#^	4.02 (0.10)	3.72 (0.17)	3.93 (0.12)	4.34 (0.12)	4.03 (0.32)	**0.002**
Hemoglobin (g/dL)	14.09 (0.09)	13.99 (0.13)	14.25 (0.15)	14.06 (0.13)	13.53 (0.36)	0.23
Platelets^#^	236.28 (3.69)	225.12 (4.52)	229.92 (4.41)	251.78 (8.73)	230.49 (21.29)	**0.002**
Albumin (g/dL)	4.29 (0.02)	4.42 (0.04)	4.32 (0.02)	4.15 (0.03)	4.34 (0.10)	**<0.0001**
Sodium (mmol/L)	139.06 (0.12)	139.20 (0.20)	139.19 (0.15)	138.83 (0.27)	139.29 (0.34)	0.59
Glucose (mmol/L)	5.80 (0.12)	5.54 (0.18)	5.52 (0.10)	6.31 (0.26)	4.98 (0.15)	**0.005**
Mortality						**0.06**
No	620 (89.60)	192 (92.05)	204 (85.26)	210 (93.24)	14 (73.48)	
Yes	80 (10.40)	22 (7.95)	32 (14.74)	22 (6.76)	4 (26.52)	

Continuous variables were summarized using mean (standard deviation), while categorical variables were presented as counts (percentages). One-way ANOVA test was applied to assess intergroup differences in continuous variables, and chi-square tests was employed to evaluate differences in categorical variables. BMI, body mass index; DM, diabetes mellitus; IFG, impaired fasting glycemia; IGT, impaired glucose tolerance; CKD, chronic kidney disease; COPD, chronic obstructive pulmonary disease; LTBI, latent tuberculosis infection; TST, tuberculin skin test; QFT-GIT, QuantiFERON-TB Gold In-Tube; WBC, white blood cell; TB: tuberculosis. # 1000cell/μL. NA, not acquire. Statistically significant differences (*p* < 0.05) were highlighted in bold.

Conversely, no statistically significant differences were observed (all *p* > 0.05) among different BMI groups regarding age, gender, marital status, alcohol consumption, smoking status, presence of asthma, COPD, mortality rate, monocyte count, and serum sodium levels.

### Underweight was associated with increased mortality in LTBI participants

**Figure 2:**
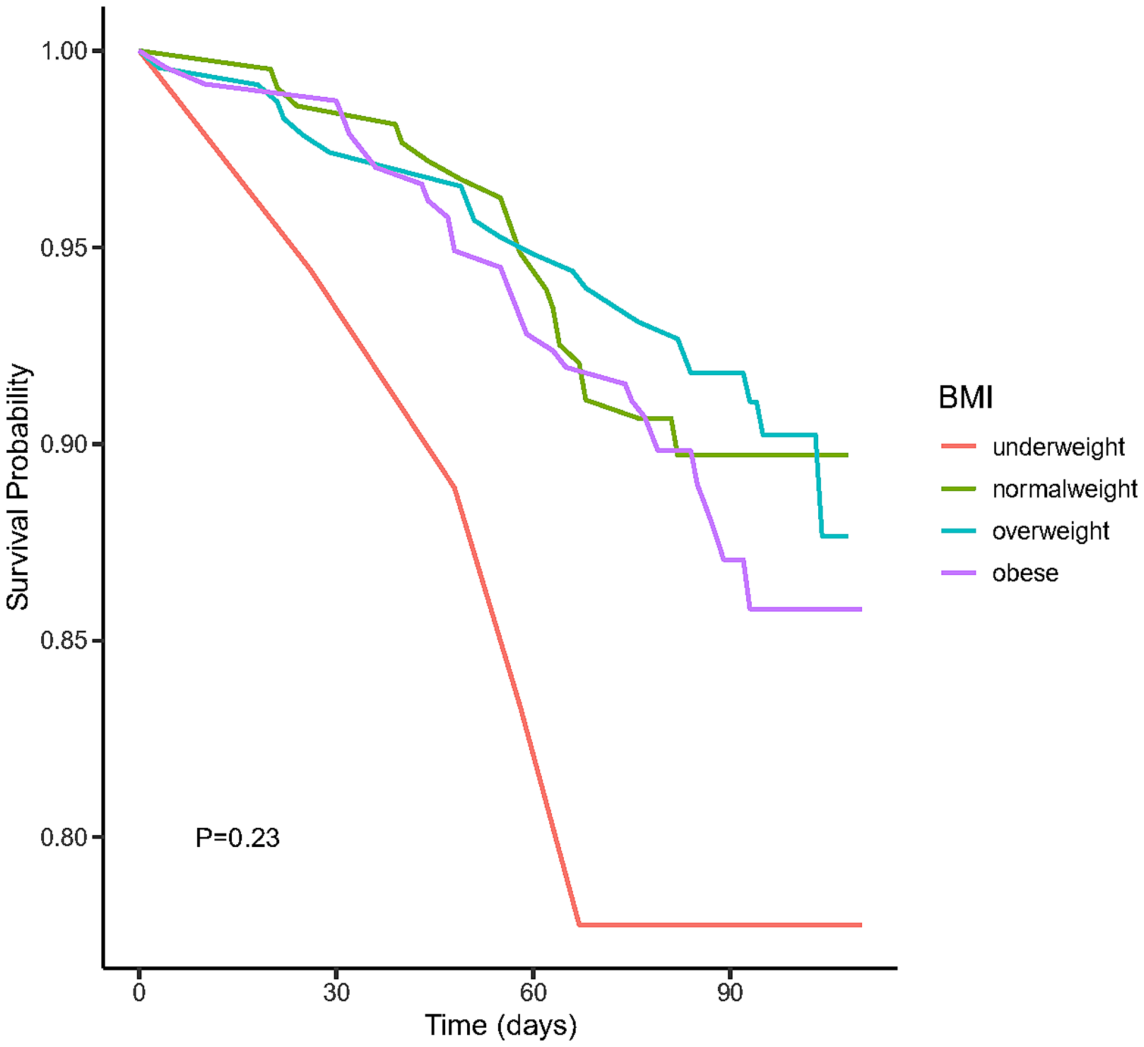
The Kaplan–Meier survival curves for distinct BMI groups.

We plotted the Kaplan–Meier survival curves for distinct BMI groups (Figure 2), revealing that the mortality rate did not exhibit statistically significant differences (*p* = 0.23). Moreover, we conducted an extensive examination of the relationship between different BMI categories and the mortality risk using Cox proportional hazard analysis in four distinct models (Table 2). Our findings indicated an increased mortality risk among underweight participants (HR = 2.77, 95% CI 1.06–7.22, *p* = 0.04). Conversely, both obese and overweight individuals demonstrated no significant mortality association across both crude and adjusted models (all *p* > 0.05).

**Table 2:** Association between BMI categories and all-cause mortality in adults with LTBI.

BMI categories	Crude model HR (95% CI)	Model 1 HR (95% CI)	Model 2 HR (95% CI)	Model 3 HR (95% CI)
Normalweight	Reference	Reference	Reference	Reference
Obese	1.90 (0.77, 4.70), *p* = 0.16	1.20 (0.61, 2.34), *p* = 0.60	1.20 (0.63, 2.28), *p* = 0.58	1.22 (0.68, 2.19), *p* = 0.50
Overweight	0.85 (0.35, 2.03), *p* = 0.71	0.65 (0.28, 1.51), *p* = 0.32	0.70 (0.33, 1.48), *p* = 0.35	0.62 (0.28, 1.37), *p* = 0.24
Underweight	3.92 (0.95,16.13), *p* = 0.06	**2.83 (1.04, 7.66), *p* = 0.04**	**3.26 (1.36, 7.80), *p* = 0.01**	**2.77 (1.06, 7.22), *p* = 0.04**

Crude model: No variates were adjusted.

Model 1: Age, gender, race and educational levels were adjusted.

Model 2: Age, gender, race, educational levels, DM, CKD, COPD and Asthma were adjusted.

Model 3: Age, gender, race, educational levels, DM, CKD, COPD, Asthma and lymphocyte were adjusted.

BMI, body mass index; LTBI, latent tuberculosis infection; HR, hazard ratio; CI, confidence interval; DM, diabetes mellitus; CKD, chronic kidney disease; COPD, chronic obstructive pulmonary disease. Statistically significant differences (*p* < 0.05) were highlighted in bold.

### RCS analysis of the association of BMI and all-cause mortality in LTBI participants

**Figure 3:**
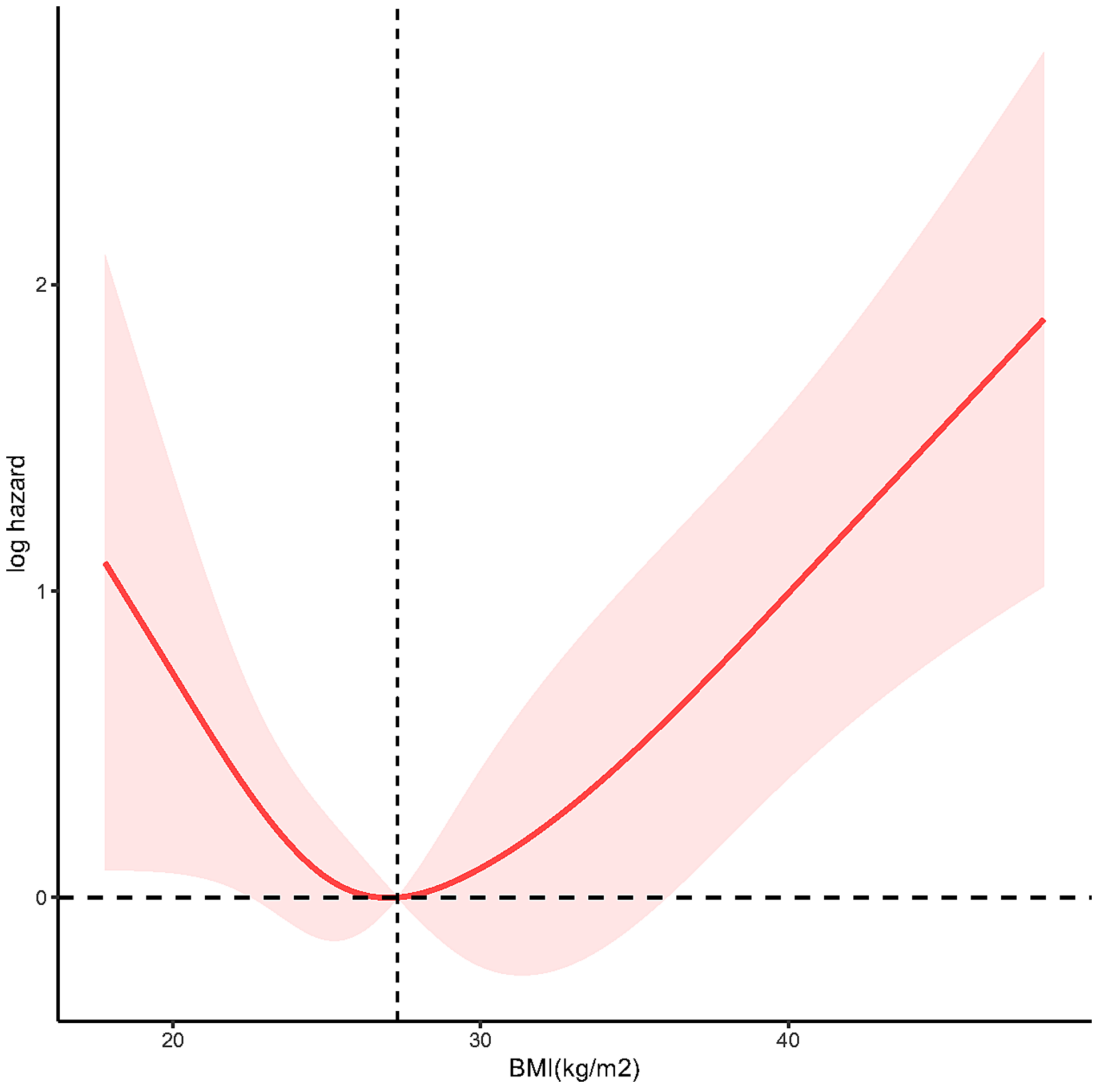
The restricted cubic spline (RCS) analysis between BMI and mortality risk of LTBI (p nonlinear = 0.0012).

To demonstrate the dose–response relationship between BMI and mortality among individuals with LTBI, we applied multivariable-adjusted RCS regression. As shown in Figure 3, the analysis revealed a U-shaped association between BMI and all-cause mortality (*p* for nonlinearity = 0.0012), with an inflection point at 27.3 kg/m^2^.

## Discussion

In this study, we enrolled 700 participants with LTBI for analysis. Our findings revealed that underweight status was associated with increased all-cause mortality among adults with LTBI after adjustment for confounding factors. Conversely, no significant association with all-cause mortality was observed for either obesity or overweight. Moreover, RCS analysis demonstrated a U-shaped relationship between BMI and all-cause mortality.

While limited study has been conducted to assess the relationship between BMI and all-cause mortality among adult individuals with LTBI, our study revealed a critical finding: although baseline mortality rates between underweight and normal-weight LTBI individuals showed no significant difference, after adjusting for key confounders (gender, age, and comorbidities), being underweight was associated with a significantly increased mortality risk. This finding aligns with broader evidence suggesting that underweight status may negatively impact TB-related outcomes. For instance, a large population-based cohort study of over ten million subjects in Korea demonstrated that the incident rate of active TB was 2.08 times higher (95% CI 2.02–2.15) in the underweight population compared to those with normal weight (13), which, although not directly measuring mortality, suggests a pathway linking low BMI to more severe TB manifestations. However, a study involving individuals with DM revealed that underweight was not a significant risk factor for LTBI progression to active TB (OR 1.07, 95% CI 0.78–1.48) (14), highlighting the potential modifying role of comorbidities. Additionally, a multi-center study of drug-sensitive pulmonary TB patients in India found that underweight patients who did not experience BMI increase after treatment initiation were associated with increased unfavorable outcomes (adjusted OR 1.81, 95% CI 1.27–2.61) (9). Our finding that confounding factors masked the underweight-mortality association underscores their critical influence on LTBI outcomes. Studies have shown lower survival rates in LTBI patients with renal insufficiency (15), and the potential underestimation of LTBI prevalence in the elderly due to higher false-negative diagnostic rates (16) further emphasizes the complex interplay of factors affecting this population’s health trajectory. Therefore, our results demonstrate that underweight is an independent risk factor for increased mortality in LTBI patients, but this risk is only evident after accounting for the substantial confounding effects of variables like age, gender, and comorbidities.

Our study revealed a clinically significant U-shaped relationship between BMI and all-cause mortality in LTBI individuals. This underscores nutritional status as a dual-edged sword: malnutrition (low BMI) and over nutrition (high BMI) independently threaten LTBI outcomes. Malnutrition can result from TB itself due to inflammation-related cachexia, anorexia, and malabsorption (17). Additionally, malnutrition can impair resistance to infection. The underlying mechanisms between underweight and poor TB infection outcomes are multifaceted. Firstly, underweight signifies inadequate nutrient intake and malnutrition, which can compromise the immune system by causing various degrees of immunodeficiency, decreased the ability of mobility, chemotaxis, attachment, and macrophage ingestion (18). It can also decrease the production of cytokines that regulate adaptive immunity against mycobacterial tuberculosis infection (19), and elevate the risk of progressing from latent infection to active disease (20). Furthermore, animal models demonstrated that imbalanced dietary nutrients can disrupt intestinal homeostasis, alter gut microbial ecology, and increase susceptibility to infection, leading to intestinal inflammation and diarrhea, further contributing to morbidity and mortality (21). Secondly, underweight individuals may have limited access to healthcare resources (22), including TB testing, diagnosis, and treatment (23), which can delay identification and management of TB infection, subsequently elevating mortality risk. Moreover, underweight individuals often have more comorbid conditions that further compromise their immune function (24, 25), heightening vulnerability to LTBI reactivation and mortality. Additionally, undernutrition reduces quality of life and economic productivity (26). Therefore, underweight individuals, particularly in low-income settings, may encounter challenges in accessing proper nutrition, healthcare, and living conditions, collectively contributing to both underweight and increased mortality risk. Which indicating that proactive nutritional support is essential for underweight LTBI individuals.

Paradoxically, our U-shaped curve inflection point fell within the overweight range, suggesting that obesity may be associated with decreased mortality. This aligns with previous studies. In a large population-based longitudinal study in Korea reported that obesity linked to lower TB mortality (27). Animal models fed a medium-fat diet have shown reduced bacterial burden and increased activation of immune cells (28). Surprisingly, in the cox regression model of our study, we did not find a significant difference in TB infection mortality between individuals with normalweight and those classified as obese. It’s important to note that our study focused on all-cause mortality rather than specific cause mortality, which may be confounded by patients’ underlying conditions. Additionally, other factors beyond weight status might play a more significant role in influencing tuberculosis outcomes.

Our study boasts several strengths. Firstly, it draws upon data from NHANES, a nationally representative population-based sample, ensuring robust sample selection and size. Secondly, we took measures to control for confounding variables, thereby enhancing the reliability of our findings. Nonetheless, our study is not without limitations. Despite our adjustments for potential covariates, we cannot completely eliminate the possibility of confounding, such as the proportions of participants progressing from latent to active TB and macronutrient supplementation. Moreover, malnutrition (29) or high BMI (30) may influence QFT-GIT test results, leading to the possible exclusion of participants with false-negative results thus some participants with false negative QFT-GIT results. Due to constraints within the mortality data, our analysis focused on all-cause mortality. Future studies should investigate TB-related mortality specifically when more data becomes available. Besides, the relatively small sample size in the underweight group reduces statistical power, increases the susceptibility of the point estimate (HR) to instability from individual cases, widens CI and limit subgroup analysis, future research must prioritize enrolling sufficient numbers of underweight individuals with LTBI to confirm this finding. Furthermore, the NHANES database exclusively represents the US population, limiting the generalizability of our results to a global context.

## Conclusion

Our findings revealed that underweight was associated with increased mortality in adults with TB infection after adjusting confounding factors, whereas no significant mortality association was observed in either obese or overweight individuals. Moreover, the utilization of RCS analysis unveiled a U-shaped correlation between BMI and all-cause mortality.

## Data Availability

The datasets presented in this study can be found in online repositories. The names of the repository/repositories and accession number(s) can be found at: https://www.cdc.gov/nchs/nhanes/?CDC_AAref_Val=https://www.cdc.gov/nchs/nhanes/index.htm.

## Footnotes

^1^ https://www.cdc.gov/nchs/nhanes/

^2^ https://www.cdc.gov/nchs/ndi/
